# *QuickStats:* Percentage* of Adults Aged ≥18 Years Who Rarely or Never Get the Social and Emotional Support They Need,^†^ by Sex and Disability Status^§^ — National Health Interview Survey,^¶^ United States, 2021

**DOI:** 10.15585/mmwr.mm7247a4

**Published:** 2023-11-24

**Figure:**
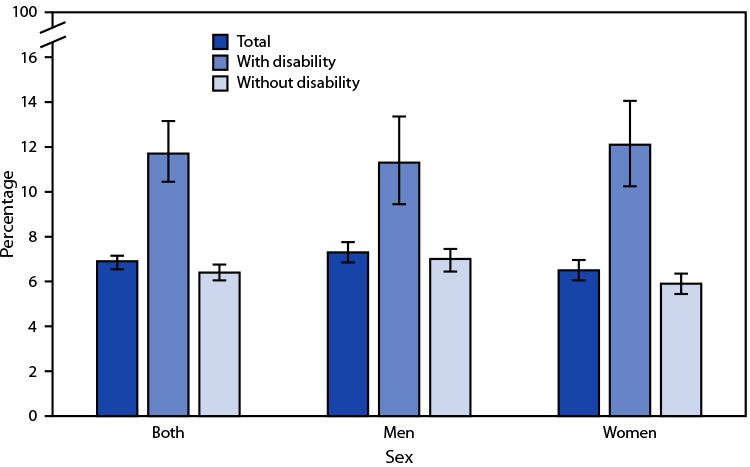
In 2021, 6.9% of adults aged ≥18 years rarely or never got the social and emotional support they needed. Overall, the percentage was higher among those with a disability (11.7%) than among those without disability (6.4%). Among men, 11.3% of those with disability rarely or never got needed support, compared with 7.0% of those without disability. Among women, 12.1% of those with disability rarely or never got needed support, compared with 5.9% of those without disability. The percentage of women and men with disability who rarely or never got the support needed was similar but was higher for men compared with women among those without disability.

**Source:** National Center for Health Statistics, National Health Interview Survey, 2021. https://www.cdc.gov/nchs/nhis/index.htm

